# Association between coronary artery calcium and all-cause mortality: A large single-center retrospective cohort study

**DOI:** 10.1371/journal.pone.0276659

**Published:** 2022-10-26

**Authors:** Mu-Cyun Wang, Che-Chen Lin, Hsiu-Yin Chiang, Hung-Lin Chen, Hsiu-Chen Tsai, Wen-Yuan Lin, Hung-Chi Ho, Chin-Chi Kuo

**Affiliations:** 1 Department of Family Medicine, China Medical University Hospital, Taichung, Taiwan; 2 School of Medicine, College of Medicine, China Medical University, Taichung, Taiwan; 3 Department of Geriatrics and Gerontology, National Taiwan University Hospital Hsin-Chu Branch, Hsinchu, Taiwan; 4 Big Data Center, China Medical University Hospital, Taichung, Taiwan; 5 Division of Cardiovascular Medicine and General Medicine, Department of Internal Medicine, China Medical University Hospital, Taichung, Taiwan; 6 Division of Nephrology, Department of Internal Medicine, China Medical University Hospital, Taichung, Taiwan; University of Otago, NEW ZEALAND

## Abstract

**Objective:**

Previous studies have revealed that coronary artery calcium is related to cardiovascular diseases and mortality. However, most studies have been conducted in Western countries and have excluded patients with pre-existing heart disease. We investigated the association between coronary artery calcium (CAC) and all-cause mortality in an Asian cohort and in subgroups stratified by age, sex, smoking, obesity, diabetes, cardiovascular disease, blood pressure, and biochemical parameters.

**Methods:**

We conducted a retrospective cohort study on 4529 health examinees who underwent multidetector computed tomography in a tertiary medical center in Taiwan between 2011 and 2016. The mean follow-up was 3.5 years. Cox regression was used to estimate the relative hazards of death. Stratified analyses were performed.

**Results:**

The all-cause mortality rates were 2.94, 4.88, 17.6, and 33.1 per 1000 person-years for CAC scores of 0, 1–100, 101–400, and >400, respectively. The multivariable adjusted hazard ratios (95% confidence intervals [CIs]) for all-cause mortality were 0.95 (0.53, 1.72), 1.87 (0.89, 3.90), and 3.05 (1.46, 6.39) for CAC scores of 1–100, 101–400, and >400, respectively, relative to a CAC score of 0. Compared with CAC ≤ 400, the HRs (95% CIs) for CAC > 400 were 6.46 (2.44, 17.15) and 1.94 (1.00, 3.76) in younger and older adults, respectively, indicating that age was a moderating variable (*p* = 0.02).

**Conclusion:**

High CAC scores were associated with increased all-cause mortality. Although older adult patients had higher risks of death, the relative risk of death for patients with CAC > 400 was more prominent in people younger than 65 years.

## Introduction

Cardiovascular disease (CVD) is the leading cause of death worldwide [1]. Classical risk factors or comorbidities of CVDs, such as hypertension [2, 3], diabetes mellitus (DM) [3, 4], hypercholesterolemia [3, 5], chronic kidney disease (CKD) [6–8], metabolic syndrome [9], smoking [3, 10], and obesity [11, 12], have been revealed to be associated with all-cause mortality. Although novel markers such as high-sensitivity C-reactive protein (hsCRP) and homocysteine have emerged for determining the risk of CVD, the predictive performance of incremental prognostic values remains controversial [13–16]. In the past three decades, computed tomography has evolved to enable the quantification of atherosclerotic plaque by measuring calcium in coronary arteries in a noninvasive manner [17]. Coronary artery calcium (CAC), a marker of subclinical atherosclerosis, has been recognized as an additive factor for predicting cardiovascular events [18, 19]. Numerous studies have demonstrated the presence of CAC as an independent predictor of coronary artery diseases [20, 21], and CAC score has been widely applied in cardiovascular risk assessment for both asymptomatic and symptomatic patients [22, 23]. Furthermore, because traditional risk factors and their combinations (e.g., Framingham Risk Score [FRS]) have predicted cardiovascular events only modestly well, adding CAC data has improved prognostic performance [20, 22]. Current guidelines recommend using CAC scores as a screening tool in individuals deemed to be at intermediate risk (FRS 10-year risk 10%–19%) [22, 24], although the prognostic value of CAC scores in women considered to be at low risk (10-year risk <10%) has also been validated [25]. The utility of CAC scores in risk stratification may also extend to younger and older patients. Younger patients with low FRSs but with CAC scores >100 had a higher risk of CVD than had older patients with intermediate FRSs but no CAC [26]. CAC scores were independently associated with all-cause mortality in adults free of known coronary heart diseases [27–29]. Two cohort studies conducted in the United States demonstrated that a CAC score >400 was more informative than traditional risk factors in predicting all-cause mortality in asymptomatic patients [30] and that its reclassification power over FRS was particularly strong in older adult patients [31]. A meta-analysis revealed that a CAC score of ≥10 predicted a composite outcome of all-cause mortality or cardiovascular events in patients with type 2 DM [32]. Despite a substantial variation in ethnic composition, CAC score consistently predicted all-cause mortality in a US cohort comprising 14,812 individuals, of whom 80% were non-Hispanic whites [33]. Other studies have reported that CAC progression was associated with all-cause mortality in patients with nonzero baseline CAC scores (i.e., presence of CAC at the beginning of the study) [34]. Although CAC and hsCRP were independently associated with all-cause mortality in a previous study, hsCRP improved the discrimination of cardiac risk only in patients without baseline CAC [35]. The aforementioned findings have mostly been based on data from Western countries, with some based on data from Asian populations [29, 33]. We aimed to replicate this fairly well-established positive association between CAC and all-cause mortality in a Taiwanese population derived from the electronic medical records (EMRs) of the largest medical center in central Taiwan. We also evaluated the moderating effects of age, sex, obesity, smoking, hypertension, diabetes, dyslipidemia, CKD, and CVD.

## Materials and methods

### Study design and participants

**Fig 1:**
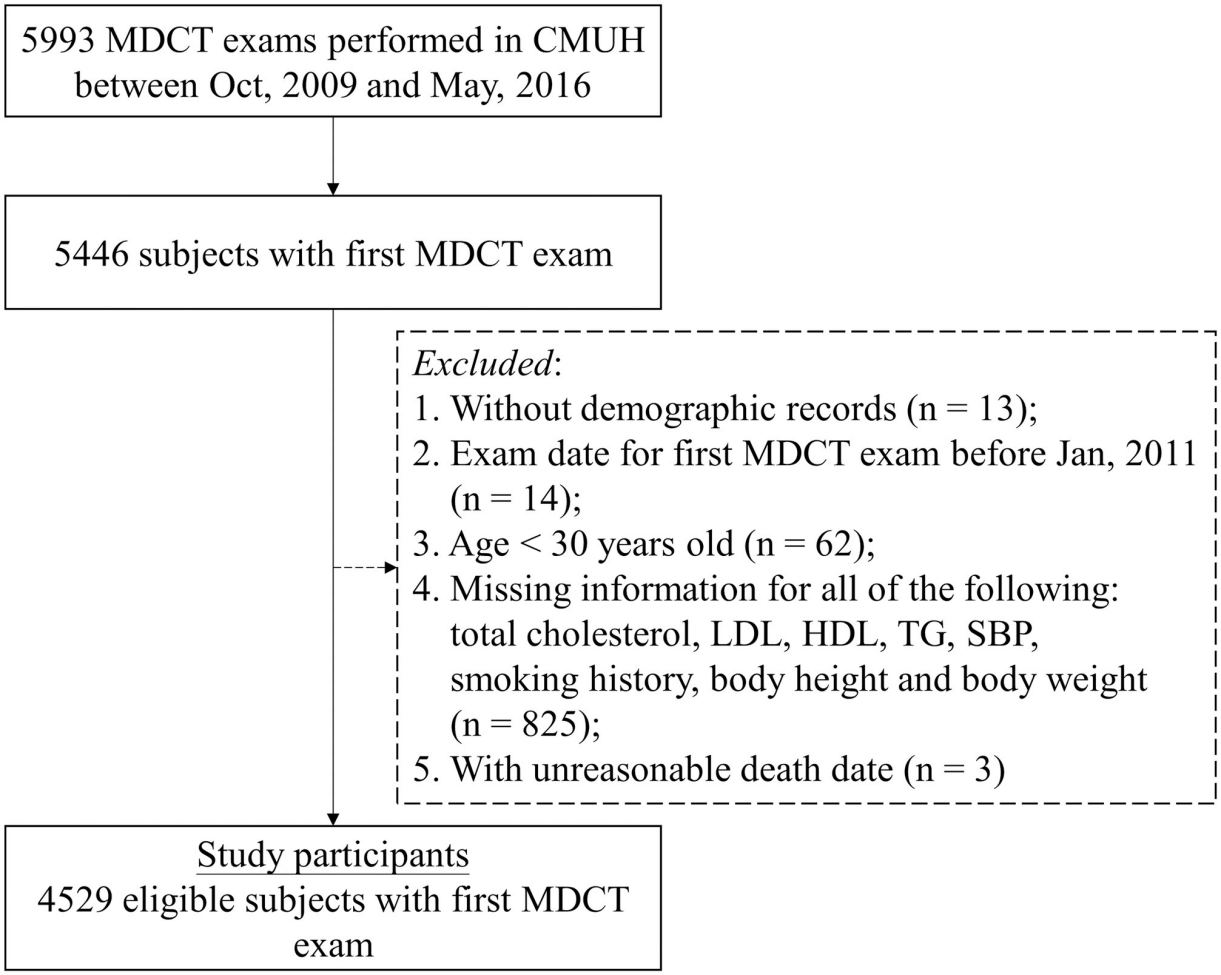
Selection process of the study population. MDCT, multidetector computed tomography; CMUH, China Medical University Hospital; CRDR, Clinical Research Data Repository; TCHO, total cholesterol; HDL, high-density lipoprotein; LDL, low-density lipoprotein; TG: triglyceride; SBP, systolic blood pressure.

This retrospective cohort study was conducted at the Health Examination Center of China Medical University Hospital (CMUH), a tertiary medical center in Taiwan. Initially, 5446 patients who underwent multidetector computed tomography (MDCT) between October 2009 and May 2016 were identified from the EMRs. Because an individual may underwent MDCT more than once during the study period, only the first MDCT report was analyzed. We excluded individuals who had their first MDCT before 2011 because of scarce data. Other exclusion criteria were age less than 30 years, lack of demographic data, completely absent anthropometric and laboratory data, and erroneous death record. The examinees could have regular (e.g., annual or biennial) health checkups at CMUH Health Examination Center or be referred by health professionals if risk factors were presented or heart disease was suspected. Patients with pre-existing CVD were also included. The flowchart of the selection process is presented in Fig 1. This study was approved with waived informed consent by the Big Data Center of CMUH and the Research Ethical Committee/Institutional Review Board of CMUH (CMUH105-REC3-068) [36, 37].

### MDCT imaging

MDCT was performed using an Aquilion One TSX-301A scanner (Toshiba, Nasu, Japan), and images were obtained at a scanning time of 0.275 ms. Approximately 56 to 64 tomographic slices with thickness of 2.5 mm were obtained between the carina and the diaphragm. Image acquisition was electrocardiographically triggered at 75% of the R-R interval. CAC was defined as a plaque of at least three consecutive pixels with attenuation of ≥130 HU. CAC scores were calculated according to the method described by Agatston [18]. All MDCT scans were reviewed by experienced radiologists.

### Demographics, comorbidities, anthropometrics, and laboratory tests

In 2017, the Big Data Center and the Office of Information Technology of CMUH established the CMUH Clinical Research Data Repository (CRDR), which carefully verifies and validates data from various clinical sources to unify trackable patient information generated during the healthcare process. Between January 1, 2003, and December 31, 2016, the CMUH-CRDR documented trackable clinical information of 2,660,472 patients who had sought care at CMUH [37]. We collected demographic data, anthropometric measurements, laboratory tests, and comorbidities relevant to coronary heart disease and deaths from the CMUH-CRDR. Weight and height, measured using an auto-anthropometer, were used to derive body mass index (BMI) (calculated as weight in kilograms divided by the square of height in meters). BMI was then categorized into four classes according to the definition released from Ministry of Health and Welfare for Taiwanese adults: underweight, BMI <18.5 kg/m^2^; normal weight, BMI 18.5–23.9 kg/m^2^; overweight, BMI 24–26.9 kg/m^2^; and obesity, BMI ≥27 kg/m^2^. Fasting plasma lipids and serum creatinine levels were determined from blood samples. Anthropometric and laboratory reports closest to but within 6 months of the dates of MDCT were used.

Individuals were classified as having CVD if they were diagnosed with coronary artery disease, peripheral vascular disease, or cerebrovascular disease in the previous year according to the coding of *International Classification of Diseases and Related Health Problems*, *Ninth Revision* (*ICD-9*), or *Tenth Revision* (*ICD-10*). DM was indicated by a history of DM or treatment with antidiabetic medications. Serum creatinine was to calculate estimated glomerular filtration rate (eGFR) using the Modification of Diet in Renal Disease equation [38]. CKD was defined as eGFR < 60 mL/min/1.73 m^2^.

### Verification of deceased status

All-cause mortality was verified by using computer linkage with a unique identification number to the national database from the Health and Welfare Data Science Center, Ministry of Health and Welfare. This database records all deaths of Taiwanese citizens, which are coded from death certificates. All individuals were followed up for a maximum of 5 years or until December 31, 2017, whichever was earlier. Follow-up was completed in 99.8% of the patients.

### Statistical analysis

CAC scores were divided into four categories—0, 1–100, 101–400, and >400—and we compared the baseline characteristics across these categories. Categorical variables were compared using the Chi-squared test, and continuous variables were compared using the Kruskal–Wallis test. The primary endpoint of this study was all-cause mortality. The mortality rate for each CAC category was calculated as the number of deaths per 1,000 person-years. We conducted both univariable and multivariable analyses by using the Cox proportional hazards model. Follow-up time was defined as the period from the date of performing MDCT to death or to the end of the study. In the multivariable model, we evaluated hazard ratios (HRs) and 95% confidence intervals (CIs) for each CAC category after adjustment for FRS risk factors, BMI, pre-existing CVD, and eGFR. Because CAC distribution may vary with other risk factors, stratified analyses and tests of interaction were conducted with respect to these variables. Because missing data (e.g., smoking status, BMI, and fasting glucose) may result in bias, we performed multiple imputations using an iterative Markov chain Monte Carlo procedure with 20 imputations and 100 iterations to impute missing values [39]. Multiply imputed data sets were combined by applying Rubin’s rule. A 5% level of significance was chosen for all analyses. All statistical analyses were performed using SAS 9.4 (SAS Institute, Cary, NC).

## Results

A total of 4529 patients were analyzed. The median age was 56.8 (interquartile range 13.5) years, and 65.3% were men. Exactly 2,148 (47.4%) patients had a CAC score of 0, and only 172 (3.8%) had CAC scores >400. Furthermore, 1,283 (28.3%) patients had been diagnosed with a CVD during the past year, and most cases were of coronary artery diseases. The median follow-up time was 3.5 (range 0.1–5.0) years. Baseline characteristics are presented in Table 1. Older people were more likely to have higher CAC scores. In addition, 91.1% of patients without CAC were less than 65 years old, whereas 58.7% of those with CAC scores >400 were 65 years and older. Men tended to have higher CAC scores, constituting 78.0% of those with CAC scores of >100. Patients with higher CAC scores had higher systolic blood pressure levels, regardless of whether they were treated. The prevalence of DM and CVD increased with CAC score. Patients with nonzero CAC scores had higher BMIs. Serum creatinine levels elevated and eGFR levels declined across increasing CAC score categories.

**Table 1:** Baseline demographics and clinical characteristics according to coronary artery calcium scores.

Variables^a^	Coronary artery calcium score					
	Overall	0	1–100	101–400	> 400	
	(*n* = 4529)	(*n* = 2148)	(*n* = 1927)	(*n* = 282)	(*n* = 172)	*p* value^b^
**Framingham risk score**						
Age, years	56.8 (49.8, 63.4)	52.9 (46.0, 58.9)	59.4 (53.3, 65.2)	64.9 (58.4, 72.2)	67.5 (59.9, 74.6)	<0.0001
Male	2957 (65.3)	1183 (55.1)	1420 (73.7)	221 (78.4)	133 (77.3)	<0.0001
Total Cholesterol, mg/dL	199 (173, 225)	201 (177, 227)	199 (171, 226)	183 (155, 211)	177 (152, 205)	<0.0001
HDL, mg/dL	45.4 (38.5, 54.4)	47.6 (40.2, 57.8)	44 (37.5, 51.9)	43.6 (36.3, 52.8)	42.8 (36.8, 50.3)	<0.0001
SBP not treated, mmHg	116 (107, 129)	113 (103, 124)	120 (112, 131)	130 (117, 136)	145 (128, 146)	<0.0001
SBP treated, mmHg	127 (116, 140)	122 (113, 134)	130 (120, 144)	131 (119, 142)	133 (121, 146)	<0.0001
Smoker	765 (28.3)	334 (25.1)	344 (30.4)	57 (37.7)	30 (35.3)	0.0005
Diabetes mellitus	325 (7.18)	68 (3.2)	176 (9.1)	42 (14.9)	39 (22.7)	<0.0001
**Cardiovascular disease**						
Coronary artery disease	1088 (24.0)	333 (15.5)	543 (28.2)	126 (44.7)	86 (50.0)	<0.0001
Peripheral vascular disease	172 (3.80)	65 (3.00)	79 (4.10)	11 (3.90)	17 (9.90)	<0.0001
Stroke	244 (5.39)	68 (3.20)	124 (6.40)	24 (8.50)	28 (16.3)	<0.0001
**Medication**						
Statin	814 (18.0)	199 (9.30)	453 (23.5)	102 (36.2)	60 (34.9)	<0.0001
Fibrate	100 (2.21)	26 (1.20)	53 (2.80)	12 (4.3)	9 (5.2)	<0.0001
**Biochemical profile**						
Creatinine, mg/dL	0.86 (0.71, 0.99)	0.81 (0.66, 0.95)	0.89 (0.77, 1.01)	0.93 (0.82, 1.07)	0.96 (0.82, 1.12)	<0.0001
eGFR, mL/min/1.73 m^2^	92.2 (80.5, 101)	96.9 (86.7, 104)	89.1 (77.7, 97.9)	82.4 (68.8, 92.8)	80.5 (64.2, 91.2)	<0.0001
**BMI, kg/m** ^**2**^	24.8 (22.6, 27.1)	24.1 (22.0, 26.4)	25.4 (23.4, 27.8)	25.6 (23.8, 27.4)	25.8 (23.1, 28.7)	<0.0001

^a.^ Presented in median (quartile 1, quartile 3) for continuous variables and number (percent) for categorical variables.

^b.^ *P* values calculated by the Kruskal–Wallis test for continuous variables and the Chi-square test for categorical variables.

**Abbreviations:** HDL, high-density lipoprotein; SBP, systolic blood pressure; eGFR, estimated glomerular filtration rate; BMI, body mass index.

Table 2 displays all-cause mortality rates, crude HRs, and multivariable adjusted HRs for different CAC categories. Overall, 91 deaths (2.0%) were recorded. The mortality rates were 2.94, 4.88, 17.6, and 33.1 per 1,000 person-years for CAC scores of 0, 1–100, 101–400, and >400, respectively. In the univariable model, CAC scores, age, DM, CVD, and eGFR were significantly associated with all-cause mortality. In the multivariable model, CAC scores, age, DM, and eGFR remained significant predictors. The association between CAC score and all-cause mortality was attenuated in the multivariable model. The HRs (95% CIs) for CAC categories 1–100, 101–400, and >400 were 0.95 (0.53, 1.72), 1.87 (0.89, 3.90), and 3.05 (1.46, 6.39), respectively. In the full model, the hazard of death rose 1.90 times for each 10-year increase in age, with other covariates held constant. Additionally, the hazards were 2.41 and 2.37 times greater in patients with DM and CKD, respectively, than in those without. Replacing categorized CAC with the continuous-scale CAC, we obtained a multivariable adjusted HR (95% CI) of 1.16 (1.05, 1.28) per unit increase in the natural log of (CAC + 1).

**Table 2:** All-cause mortality risks according to CAC and other risk factors.

Variables	Crude HR (95% CI)	*p* value	Adjusted HR (95% CI)	*p* value
**CAC**				
0	ref	-	ref	-
1–100	1.66 (0.97, 2.85)	0.07	0.95 (0.53, 1.72)	0.87
101–400	5.99 (3.18, 11.3)	<0.0001	1.87 (0.89, 3.90)	0.10
>400	11.3 (6.14, 21.0)	<0.0001	3.05 (1.46, 6.39)	0.003
**Age, years**	1.10 (1.08–1.12)	<0.0001	1.07 (1.04, 1.09)	<0.0001
**Sex**				
Female	ref	-	ref	-
Male	0.77 (0.51–1.18)	0.23	0.73 (0.44, 1.22)	0.23
**BMI, kg/m** ^**2**^				
<18.5	1.27 (0.39, 4.17)	0.70	1.49 (0.41, 5.45)	0.54
18.5–23.9	ref	-	ref	-
24–26.9	0.81 (0.46, 1.42)	0.47	0.74 (0.42, 1.32)	0.31
≥27.0	0.63 (0.31, 1.30)	0.21	0.52 (0.25, 1.09)	0.08
**Smoking history**				
No	ref	-	ref	-
Yes	1.03 (0.62, 1.70)	0.91	1.45 (0.80, 2.63)	0.22
**Diabetes mellitus**				
No	ref	-	ref	-
Yes	4.37 (2.72–7.01)	<0.0001	2.41 (1.44, 4.01)	0.0007
**SBP, mmHg**				
<140	ref	-	ref	-
≥140 or treated	2.68 (0.89, 8.12)	0.08	1.67 (0.53, 5.28)	0.38
**Total cholesterol, mg/dL**				
<200	ref	-	ref	-
≥200 or treated	0.64 (0.41, 1.01)	0.05	0.63 (0.39, 1.02)	0.06
**HDL, mg/dL**				
≥40	ref	-	ref	-
<40	1.23 (0.76, 2.01)	0.40	1.28 (0.75, 2.17)	0.37
**eGFR, mL/min/1.73 m** ^**2**^				
≥60	ref	-	ref	-
<60	7.93 (5.00, 12.6)	<0.0001	2.37 (1.40, 4.00)	0.001
**Cardiovascular disease**				
No	ref	-	ref	-
Yes	2.46 (1.63–3.71)	<0.0001	1.05 (0.66, 1.66)	0.84

**Abbreviations:** CAC, coronary artery calcium; BMI, body mass index; SBP, systolic blood pressure; HDL, high-density lipoprotein; eGFR, estimated glomerular filtration rate.

**Fig 2:**
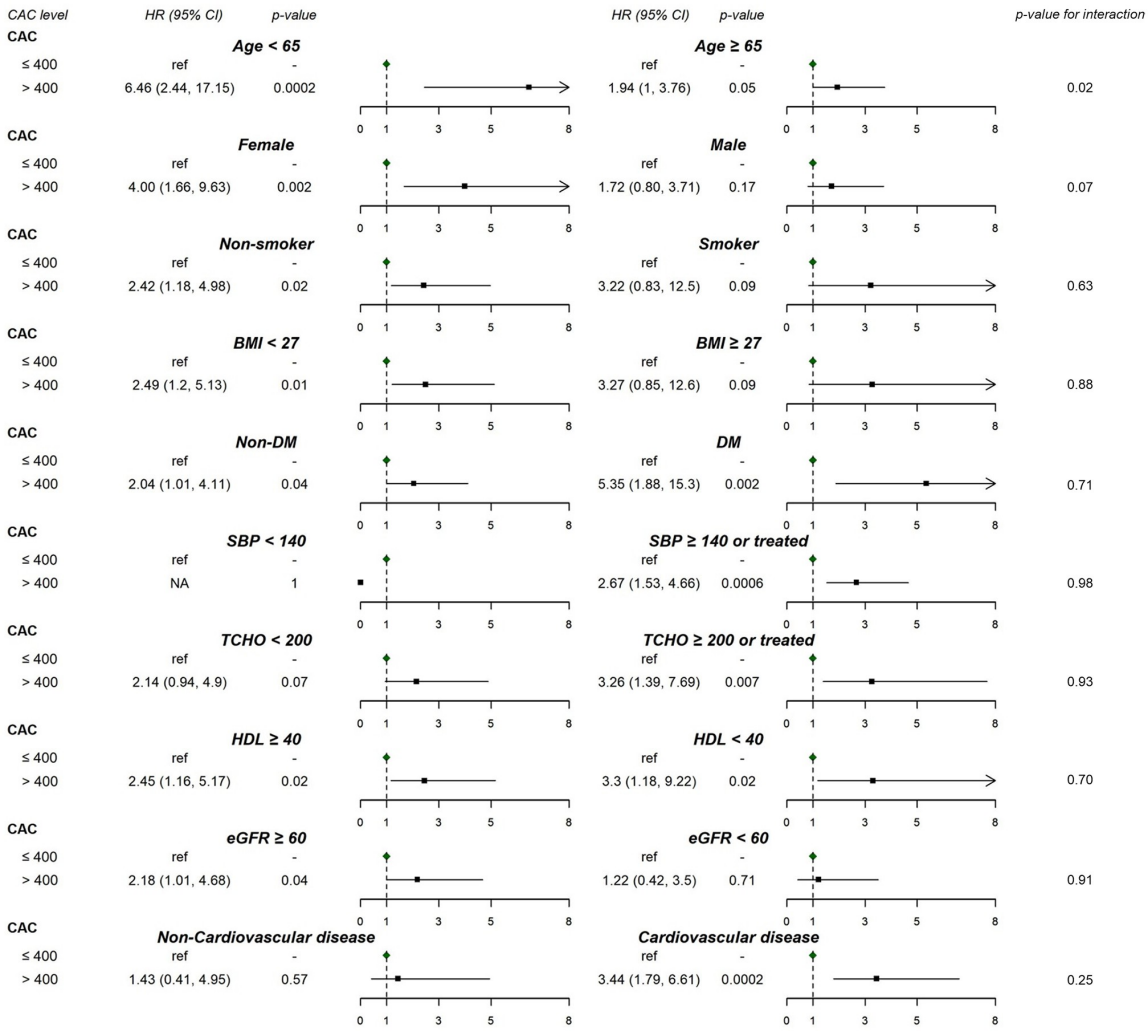
Multivariable HRs (95% CIs) of mortality risks according to CAC stratified by other variables. BMI, body mass index; CAC, coronary artery calcium; DM, diabetes mellitus; SBP, systolic blood pressure; TCHO, total cholesterol; HDL, high-density lipoprotein; eGFR, estimated glomerular filtration rate.

We further conducted stratified analyses for age, sex, smoking, obesity, DM, hypertension, dyslipidemia, CKD, and CVD. Fig 2 presents multivariable adjusted HRs (95% CIs) for CAC > 400 versus CAC ≤ 400 and tests for interaction. To avoid residual confounding from dichotomizing continuous variables, we further adjusted the continuous form of the corresponding variable within each subgroup. Although the mortality rate was much higher for people aged 65 years and older irrespective of CAC category, the association of CAC > 400 with increased mortality was stronger in younger adults. The HRs (95% CIs) for CAC > 400 versus CAC ≤ 400 were 6.46 (2.44, 17.15) and 1.94 (1.00, 3.76) in younger adults and older adults, respectively. Women with CAC scores >400 had a higher mortality rate than men with similar CAC scores, and the HR (95% CI) for CAC > 400 versus CAC ≤ 400 was 4.00 (1.66, 9.63). For non-smokers, patients without obesity, and patients without CKD, the HRs (95% CIs) for CAC > 400 versus CAC ≤ 400 were 2.42 (1.18, 4.98), 2.49 (1.20, 5.13), and 2.18 (1.01, 4.68), respectively. For patients with hypertension, hypercholesterolemia, and CVD, the HRs (95% CIs) for CAC > 400 versus CAC ≤ 400 were 2.67 (1.53, 4.66), 3.26 (1.39, 7.69), and 3.44 (1.79, 6.61), respectively. The test for interaction suggests that age was a variable moderating the association between CAC scores and mortality (*p* = 0.02). However, when we treated both CAC scores and age as continuous variables, the test for interaction had a *p* value of 0.67. This discrepancy may be due to the nonlinear relationship of CAC with all-cause mortality.

**Fig 3:**
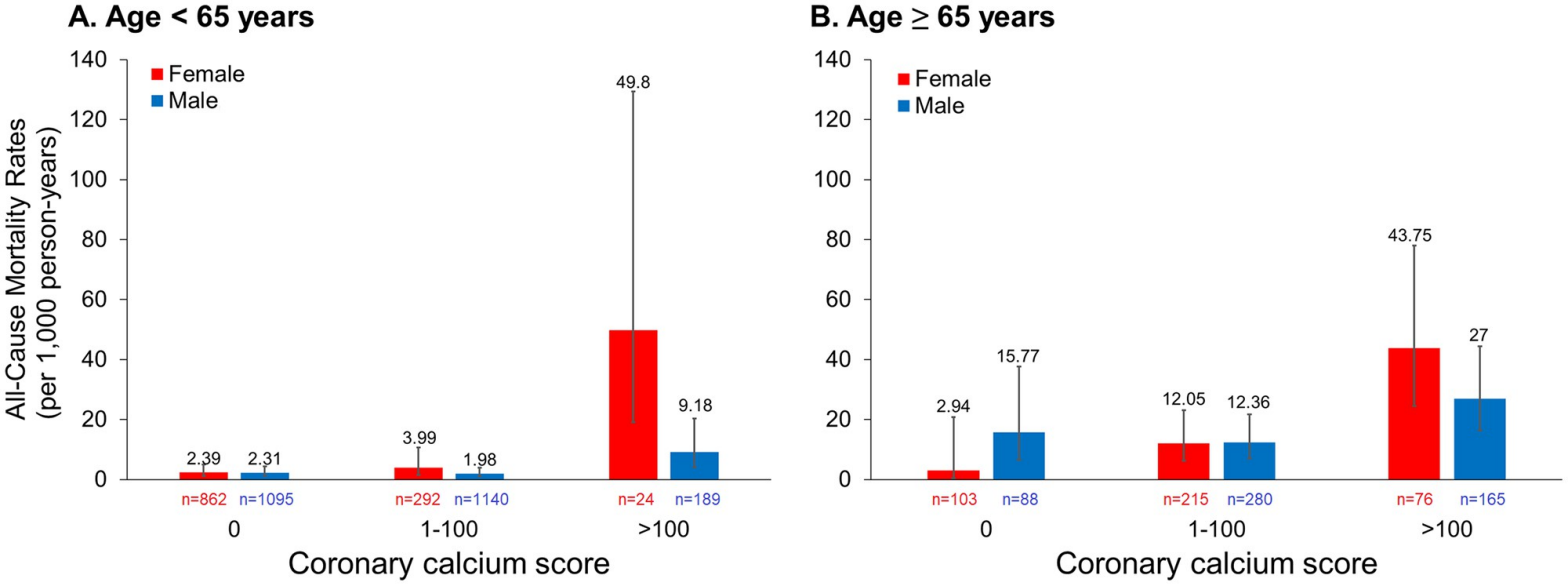
Unadjusted all-cause mortality rates according to coronary artery calcium (CAC) score by age and sex. (A) Age < 65 years and (B) age ≥ 65 years.

Fig 3 illustrates the three-factor interaction of age, sex, and CAC on all-cause mortality. We collapsed the last two CAC categories with sparse cell counts given sample size limitations. The crude mortality rate for a zero CAC was comparable between both sexes in younger patients but was much higher in men than in women as patients approached 65 years. In older adult patients, only women exhibited a steadily increasing risk as CAC levels increased, but comparable risks of death were observed in both sexes for CAC 1–100. Notably, the mortality rate was extremely high in young women with CAC > 100; however, this finding may be less accurate due to the wide CI owing to rare mortality events. Table 3 shows the multivariable-adjusted HRs of all-cause mortality for different CAC categories, stratified by age and sex. Among all age-sex combinations, CAC > 100 was significantly associated with higher mortality only in women under 65 years, compared with CAC = 0 although we did not observe a meaningful difference in sex. The result was consistent if CAC categories were replaced by the continuous form, with the adjusted HR (95% CI) being 1.29 (1.03, 1.62) per unit increase in natural log of (CAC+1) for women under age 65 (not shown). Further clinical research is required to verify whether young and middle-aged women are more vulnerable to developing cardiovascular events from high CAC burden.

**Table 3:** Mortality risks presented in HR (95% CI) according to coronary artery calcium score, stratified by subgroups of age and sex.

Stratum	*N*	deaths	Coronary artery calcium score			*p* value for interaction^a^
			0	1–100	> 100	
**Age <65 years**						
Female	1178	15	ref	1.18 (0.29, 4.73)	7.26 (1.31, 40.1)	0.403
Male	2424	23	ref	0.65 (0.24, 1.78)	3.02 (0.92, 9.84)	
**Age ≥65 years**						
Female	394	21	ref	3.01 (0.36, 25.4)	5.59 (0.63, 49.7)	0.293
Male	533	32	ref	0.67 (0.23, 2.01)	1.22 (0.41, 3.60)	

^a^Interaction between coronary calcium score and sex under age group. Model adjusted for age, body mass index, smoking, diabetes mellitus, systolic blood pressure, total cholesterol, high-density lipoprotein, estimated glomerular filtration rate and cardiovascular disease.

## Discussion

Our findings revealed a positive association between CAC and all-cause mortality in a middle-to-old-aged Taiwanese cohort. Although CAC > 400 was consistently associated with higher mortality across all age categories, this association was more apparent in younger adults. Our study provides the first Taiwanese data from the real-world practice settings which supports the potential prognostic value of CAC assessed by MDCT.

Coronary calcification is considered as a subclinical atherosclerotic process, and its association with cardiovascular events and deaths has been well validated [20, 28, 29]. Our results, consistent with those of some studies on Asian populations, support a positive association between CAC and all-cause mortality [40, 41]. The cut-point of CAC scores have varied across studies. A three-center (Torrance, CA; Columbus, OH; Nashville, TN) study was conducted among 44,052 middle-aged patients free of baseline coronary heart diseases in the United States, with a mean follow-up of 5.6 years. According to the study, the mortality rates for CAC scores 0, 1–10, and >10 were 0.87, 1.92, and 7.48 per 1000 person-years, and adjusted HRs (95% CIs) for CAC = 1–10 and CAC > 10 versus CAC = 0 were 1.99 (1.45, 2.75) and 4.08 (3.30, 5.04), respectively [27]. In the CAC Consortium, a multicenter cohort study of 66,363 asymptomatic adults in the United States with a mean follow-up of 12 years, a CAC score of 1–10 was associated with an increased risk of all-cause and CVD mortality only in patients less than 40 years [42]. In the present study, however, individuals with CAC 1–100 did not have a significantly greater mortality risk than those without CAC. Although our study and the two aforementioned studies comprised individuals of comparable ages, our study had smaller sample size, and a median follow-up of 3.5 years may be too short to achieve sufficient statistical power. Therefore, the results of our study must be interpreted with caution.

Our study demonstrated that age was a moderating variable, because the positive association between CAC and all-cause mortality was more prominent in younger adults. Tota-Maharaj et al. reanalyzed the aforementioned three-center cohort and observed a similar interaction between a CAC score of >400 and age in all-cause mortality [43]. In the CAC Consortium, a CAC score of >10 was associated with a higher risk of all-cause mortality in all age strata, with HR (95% CI) of 2.90 (1.36, 6.21), 1.81 (1.37, 2.38), 1.77 (1.47, 2.13), 1.43 (1.19, 1.72), and 1.80 (1.44, 2.25) in those <40, 40–50, 50–60, 60–70, and ≥70 years, respectively. These associations were significantly weaker in higher age strata (*p* for interaction = 0.002) [42]. Younger adults with advanced CAC may have a more aggressive form of atherosclerosis. Another reason may be that collateral vessels are not yet developed in younger patients with coronary artery disease [44]. Consequently, young adults may be more vulnerable to the CAC burden, and the CAC score may have greater value in the risk assessment of young adults.

We also observed that a CAC score of >400 was associated with somewhat higher all-cause mortality in women than in men (*p* for interaction = 0.07). Raggi et al. conducted a cohort study consisting of 35,383 patients free of coronary artery diseases who were referred by primary physicians owing to presence of risk factors [31]. They reported a higher mortality risk in men than in women in all age deciles and CAC categories. Sex differences in survival were also more prominent in younger deciles with higher CAC scores, as an interaction between sex and CAC was observed within age strata (all *p* for interaction < 0.0001). In a multiethnic study by Nakanishi et al., which recruited 13,092 individuals with similar age and sex distributions to those of the present study and had a much longer follow-up of 11 years, the association between CAC and mortality was stronger in individuals aged <75 years, but not did not differ by sex [29]. Our study was not consistent with that of Raggi et al. in that men with detectable CAC had a lower risk than corresponding women. Notably, men were slightly younger than women in our study. For individuals with no CAC, the mortality risks for both sexes were similar in those aged <65 years, and men exhibited a higher risk when they attained 65 years (Fig 3). Nakanishi et al. reported that mortality risk increased progressively with CAC levels in both sexes, but was higher in women aged <75 years with CAC > 400, which is consistent with our results. In their study, women were more likely to have a family history of CVD, but our study lacks this information.

Shaw et al. conducted a multicenter cohort study enrolling 63,215 asymptomatic individuals in the United States, with a median follow-up of 12.6 years [45]. They reported that both all-cause and CVD mortality were consistently higher in women with CAC >100 than in men with comparable CAC. On average, men have higher CAC scores and earlier development of CAC; thus, equal CAC scores in both sexes may not confer the same risk of clinical outcomes. Shaw et al. also observed that given the same CAC stratum, women had fewer but larger calcified lesions than men, which could lead to poorer prognosis [45]. Plank et al. conducted a matched cohort study of 1050 patients with a mean follow-up of 5.6 years and demonstrated that men had significantly more calcified plaques, whereas women had more mixed and non-calcified plaques. Hence, there may be excess risk of atherosclerosis in women beyond their CAC scores [46].

The strength of our study includes its large hospital-based population, inclusion of patients with and without CVD, standardized protocol of MDCT examination in a single center, and complete clinical data extracted from the EMRs [37]. Our study, however, had several limitations. First, although numerous participants underwent MDCT as part of their regular checkup, they may still have had conventional risk factors more frequently than the general population as they received health checkup in a tertiary hospital. Indeed, patients with established risk factors are more likely to undergo CAC screening. This explains the higher prevalence of hypertension, diabetes, and dyslipidemia in our cohort compared with that of the national survey [47–49]. Thus, our results may not be applicable to the general population. Second, residual confounding cannot be completely excluded because of lack of sociodemographic information such as education, exercise, and family history of premature CVDs. Finally, only 91 deaths were recorded owing to the relatively small sample size and short follow-up period. The stratified analysis may be underpowered to detect a true difference. We collapsed the top two CAC categories in the three-factor interaction analysis, yet wide CIs were still obtained for younger and older women (Table 3). We therefore did not specifically analyze for CVD deaths due to rare events (less than 30) during the short follow-up, although CVD mortality ought to be more relevant to coronary artery calcification.

## Conclusions

CAC was associated with all-cause mortality in Taiwanese adults, independent of other well-established cardiovascular risk factors. This relationship was particularly apparent in younger adults, with age being a moderating variable. Future directions include conducting a well-designed prospective study to establish and verify a prognostic model that comprehensively incorporates calcium scores and other risk factors. Analysis for cause-specific mortality is also warranted to promote a better understanding of the mediating mechanisms of subclinical atherosclerotic process and to evaluate whether patients at risk would benefit from intervention, beyond cardiovascular deaths.
